# Competitive Intensity Modulates the Pain Empathy Response: An Event-Related Potentials Study

**DOI:** 10.3389/fpsyg.2018.01854

**Published:** 2018-10-01

**Authors:** Pinchao Luo, Yu Pang, Beibei Li, Jing Jie, Mengdi Zhuang, Shuting Yang, Xifu Zheng

**Affiliations:** ^1^School of Psychology, Center for Studies of Psychological Application, and Guangdong Key Laboratory of Mental Health and Cognitive Science, South China Normal University, Guangzhou, China; ^2^School of Education and Psychological Science, Sichuan University of Science and Engineering, Zigong, China

**Keywords:** pain, empathy, competitive intensity, ERP, N2, LPP

## Abstract

Previous studies have widely reported that competition modulates an individual’s ability to empathize with pain experienced by others. What remains to be clarified, however, is how modulations in the intensity of competition might affect this type of empathy. To investigate this, we first used a Eriksen Flanker task to set different competitive intensity context (high competitive intensity, HCI; medium competitive intensity, MCI; low competitive intensity, LCI). Then we used a recognition task as a competitive task, in which we recorded event-related potentials (ERP) while participants viewed static images of body parts in painful and non-painful situations. Participants were informed that both sets of images depicted an opponent that they were required to play against in the recognition task that varied in levels of competitive intensity according to condition (HCI, MCI, and LCI). We observed an early N2 differentiation between pain and no-pain stimuli over the frontal area under MCI and LCI conditions, but this was not detected under HCI condition. Moreover, we observed a pattern of pain and no-pain differentiation for the late LPP over the frontal and centro-parietal regions under HCI, MCI, and LCI condition. As the pain empathy response is indexed by pain and no-pain differentiation, these results indicate a down-regulation of pain empathy response attributable to a high level of competition. With its very early onset, this effect appears to inhibit bottom-up processing of the ability to perceive pain experienced by an opponent. Our results provide neuroscientific evidence for a deficit in early automatic arousal in response to the pain of the opponent under the influence of high competitive intensity.

## Introduction

Pain empathy has been defined as the ability to understand and experience the painful feelings of another individual through cognitive/evaluative and affective processes (Jackson et al., 2006; Decety et al., 2010; Bernhardt and Singer, 2012; Gao et al., 2017). This ability enables us to understand the pain of others, to motivate prosocial behavior, and to reduce racial biases (Coke et al., 1978; Batson et al., 2002; Burgay et al., 2003; Todd et al., 2011; Graaff et al., 2018; Travis et al., 2018). Due to its significant role in social interaction, pain empathy has become an intense area of research in psychology and neuroscience (Sassenrath et al., 2016; Yang et al., 2017). Recently, a large body of studies have investigated whether and how pain empathy is affected by social factors such as sense of fairness (Singer et al., 2006), monetary reward (Guo et al., 2012; Goerlich et al., 2016), affective preference (Wang et al., 2014; Yang et al., 2014), social distance (Zaki, 2014; Wang et al., 2016), as well as competition and cooperation (Cui et al., 2016).

One interesting aspect of this research is that people would regulate their pain empathy response to others when operating within a context that is competitive. The effect of competition on empathy for pain remains a controversial issue. For instance, some studies have found that relative to in-group members, participants experience less empathy toward out-group members when the two groups are in a competitive relationship (Cikara et al., 2011, 2014). Other studies have observed that people are friendlier, more helpful, and more willing to respond to others in cooperation, but more aggressive, less helpful, and less willing to empathize with their opponents (Leach et al., 2003; Deutsch, 2015; Ouwerkerk et al., 2016; De Vos et al., 2016; Suleiman et al., 2018). However, affective responses to the opponent’s pain in competitive context were characterized not only by less empathy but also by increased counter-empathic responses: schadenfreude (Cikara et al., 2014). An ERP study conducted by Yamada et al. (2011) compared empathic responses to the cooperative coplayer and the competitive coplayer. The results showed that the affective expression of the coplayer presented to the participants would induced congruent empathic responses under cooperative condition, while incongruent counter-empathetic responses occurred under competitive condition (Yamada et al., 2011). Another recent ERP study conducted by Cui et al. (2016) compared brain response when participants passively viewed images depicting the hands or feet of anonymous individuals in painful and non-painful situations. Importantly, viewing took place within both competitive, and cooperative contexts. That is, alongside but unrelated to the images, participants played a game which they were instructed to play competitively or cooperatively with a partner. They found that viewing others in pain elicited significantly larger P3 amplitudes than the non-pain-related pictures, however this effect was only observed within the competitive context and not within the cooperative context. This result indicated that the participants were more responsive to other’s pain in a competitive context than in a cooperative context due to the threatening atmosphere induced by competitive context.

In these previous studies mentioned above, the influence of competitive social context on empathy was investigated by comparing empathy response in competitive and cooperative context (Koban et al., 2012; De Dreu and Kret, 2016; Lee et al., 2018) or by recording empathic response only in competitive context (Yamada et al., 2011). The intensity of competitive context was not taken into account. However, competition has a dynamic continuous structure ranging in intensity from weak to strong (Li et al., 2012) in real life. Previous studies have found that differences in competitive intensity represent different levels of threat (Ibáñez et al., 2011; Brankley and Rule, 2014). As a result, these variations in competitive intensity have differential effects, such that a higher competitive intensity context would create a more negative and threatening atmosphere. Accordingly, schadenfreude but not empathic response to the opponent’s pain is more likely to occur. What is currently unknown is whether the empathic response to pain and non-pain stimuli is modulated as a function of differing intensities of competition context.

It is suggested that empathy involves both an early automatic component characterized by emotional sharing (bottom-up processing) and a late controlled component characterized by cognitive evaluation (top-down processing) (Decety and Lamm, 2006; Xiang et al., 2018). Evidences from ERP studies have shown that the temporal dynamics of empathy for pain consists of an early affective arousal component (N1/N2) followed by a late cognitive reappraisal and regulation component (P3/LPP) (Luck and Hillyard, 2000; Fan and Han, 2008; Han et al., 2008; Cheng et al., 2017; Decety et al., 2017). Functional magnetic resonance imaging studies have also demonstrated engagement of the anterior insula (AI), anterior cingulate cortex (ACC), brain stem, and cerebellum during observation of other people in painful situations (Singer et al., 2004; Jackson et al., 2005; Cheng et al., 2007; Gu and Han, 2007; Lamm et al., 2007, 2011; Walter et al., 2016; Lee et al., 2018). It also remains to be clarified whether bottom-up and top-down information processes of pain empathy are associated with these differences in competitive intensity.

To investigate this, we first used a Eriksen Flanker task to set different competitive intensity context. Given that competition is rooted in evaluation, reward, wins and losses (Amabile, 1996), different competitive intensity contexts could be implemented by manipulating the possibility of winning (Li et al., 2012). Consistent with the research paradigm of Li et al. (2012), we manipulated the variable of competitive intensity by varying the probabilities of winning as a function of condition (HCI condition, 10% probability; MCI condition, 50% probability; and LCI condition, 90% probability). Then we used a recognition task as competitive task, in which we recorded event-related potentials (ERP) while participants viewed static images of body parts in painful and non-painful situations. Participants were informed that both sets of images depicted an opponent that they were required to play against in the judgment task that varied in levels of competitive intensity according to condition (HCI, MCI, and LCI).

According to previous studies (Decety et al., 2010; Luo et al., 2015), the pain empathy response is indexed by pain and no-pain differentiation. The early empathic N2 component is supposed to be automatic, bottom-up driven and more dependent on the context or characteristic of stimulus (Fan and Han, 2008). In addition, compared with low competitive intensity, high competitive intensity represented a greater threat to the possibility of winning (Diehl and Stroebe, 1991), because the success of participants’ opponent would reduce the possibility of their own success when competing for the same goal. Therefore, we expected that N2 differences between pain/no-pain stimuli in the early empathic response could not be found only under HCI condition, as high competitive context would hinder automatic empathic response. The late empathic LPP component is supposed to be controlled-top-down driven. As we know, empathy induces prosocial behavior and is widely appreciated by the society (Coke et al., 1978; Batson et al., 2002). Thus, we anticipated that the three conditions would show LPP differences between pain/no-pain stimuli, as top down mechanisms would reappraise the stimuli and regulate the empathic response to conform to social expectations.

## Materials and Methods

### Participants

Forty-seven college students (23 females, 24 males) aged 19–24 years (mean age, 21.3 years; SD 2.3 years) were enrolled in the study. Two participants (1 female, 1 male) were excluded from data analysis because of intensive head movements during electroencephalographic (EEG) recording (over 15% bad epochs). All participants were right-handed, with normal or corrected to normal vision, and reported no history of neurological, brain injuries, or developmental disabilities. Each participant signed an informed consent form and received monetary compensation for the experiment. The study was approved by the Academic Committee of South China Normal University. The experimental procedure met the standard of ethical standards of the Declaration of Helsinki (British Medical Journal Publishing Group, 1996).

### Visual Stimuli

Similar to those in previous ERP studies (Fan and Han, 2008; Decety et al., 2010), visual stimuli of judgment task in ERP session consisted of 60 digital color pictures showing a person’s hand or foot in painful or non-painful situations (30 each). The accidents in the pictures depicted everyday life scenarios. Pain pictures included situations such as a hand trapped in a door or cut by scissors. Each pain picture was matched with a non-pain picture that showed similar events in the same contexts, but without the nociceptive component. All of them had the same size of 9 × 6.76 cm (width × height) and were of 100 pixels per inch. Each picture was presented at the center of a 17-in. color monitor against a white background, subtending a visual angle of 2.86 × 2.29 at a viewing distance of 100 cm. Besides that, the visual stimuli used in the Eriksen Flanker task were four strings (“<<<<<”, “>>>>>”, “<<><<”, and “>><>>”), which were similar to those in previous studies (Cui et al., 2016).

### Experimental Procedure

**FIGURE 1:**
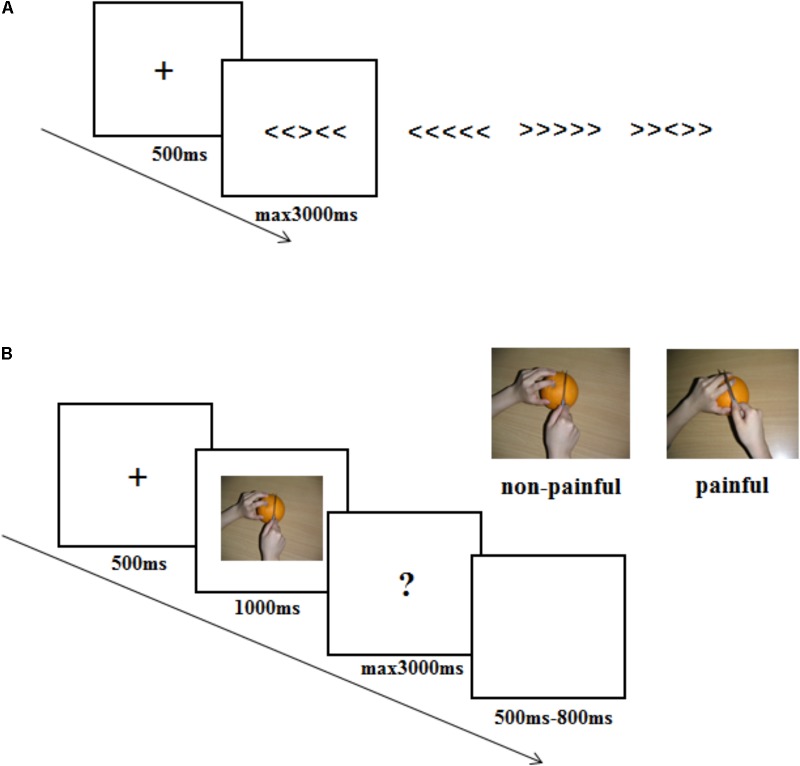
Illustration of the stimuli and experimental procedure used in the current study. **(A)** stimuli used and timing of events in one trial in the Eriksen Flanker task. **(B)** stimuli used and timing of events in one trial in the ERP experiment procedure.

Once consent forms were signed, one experimenter pretended to be the opponent was introduced to the participant. All participants were informed that the opponent would sit in another room and play the competitive game with him/her. The experiment consisted of two parts. One was a Eriksen Flanker task and the other was an ERP session. The former was to set different competitive intensity context, while the latter was to record empathy brain response to the opponent’s pain within different competitive intensity context. The experimental procedure was shown in **Figure 1**.

The Eriksen Flanker task contained 30 flanker task trials. In each of the flanker trials, a fixation was presented on a white screen for 500 ms, followed by one of the four strings: “<<<<<”, “>>>>>”, “<<><<”, and “>><>>”. Participants were instructed to respond to the arrow in the middle of the string by pressing “F” or “J” buttons (“<” corresponded to “F” and “>” corresponded to “J”). String presentation lasted for a maximum 3000 ms until a response was given. Participants were informed that the computer would compare their results with the opponents’ results according to the reaction time and accuracy, and predict their probability of winning in the subsequent recognition task in ERP session. In actual fact, participants were randomly assigned to one of three different competitive intensity groups: HCI condition (10% probability of winning), MCI condition (50% probability of winning) and LCI condition (90% probability of winning). To ensure that the competitive intensity manipulation was successful, participants were asked to rate the competitive intensity (3-point scale: 1 = LCI, 2 = MCI, 3 = HCI) and threat level (10-point scale: 1 = no threat, 10 = big threat) that they felt once the probability of winning was announced.

In the ERP session, participants were told that the reward would depend on whether they could win in the following recognition task, in which they were asked to imagine the pictures they watched belonged to their opponents and recognize the affective response (painful or non-painful) of their opponents. If the participant had a higher accuracy and faster reaction time than the opponent, he/she could win 50 RMB. Otherwise, he/she got 0 RMB. Participants were also told that their opponents would finish the same task, with one difference: the opponents imagined the pictures they watched belonged to the participants. Participants in different competitive intensity conditions performed the same ERP procedure. ERP recordings were made up of four sessions with each containing 60 trials. The order of the trial condition (pain, non-pain) was randomized. In each trial, a black fixation against a white screen was presented for 500 ms, followed by a picture for 1000 ms. Then a question mark would remain for a maximum 3000 ms until a response was given. Here, the participants were asked to recognize the valence of the pictures (pain or non-pain) and press “F” or “J” buttons (“F” corresponded to “pain”, “J” corresponded to “no-pain”) as quickly and accurately as possible. The trial ended with a blank screen varying in duration from 500 ms to 800 ms randomly. At the end of the ERP session, participants were asked to answer the questions “The hand in the picture belongs to whom? (1 = the opponent, 2 = a stranger)” and “What are your feelings when you see the opponent in pain? (1 = unpleasant, 2 = pleasant, 3 = no feeling)”.

After ERP recording, to measure individual differences of empathy, participants were instructed to fill in the Interpersonal Reactivity Index (IRI) (Davis, 1983) including four subscales: perspective taking (PT), fantasy (FS), empathic concern (EC), and personal distress (PD).

### ERP Recording and Analysis

Electroencephalogram (EEG) data were recorded from 64 scalp electrodes mounted on an elastic cap according to the extended 10–20 system (Brain Products, Germany), with references on the left and right mastoids and a ground electrode on the medial frontal aspect. Eye blinks and vertical eye movements were monitored with electrodes located above and below the left eye. The horizontal EOG was recorded from electrodes positioned 1.5 cm lateral to the left and the right external canthi. The EEG activity was amplified at 0.01–100 Hz band-passes and sampled at 500 Hz. All electrode impedances were kept below 5 kΩ. ERPs under each condition were computed separately off-line using Brain Vision Analyzer 2.0 software (Brain Products, Germany) (Fritsch and Kuchinke, 2013). ERP waveforms were time-locked to the onset of stimuli. The average epoch was 1200 ms, including a 200 ms pre-stimulus baseline. Trials contaminated by eye movements and muscle potentials exceeding ± 100 μV at any electrode or response errors were excluded from the average.

**FIGURE 2:**
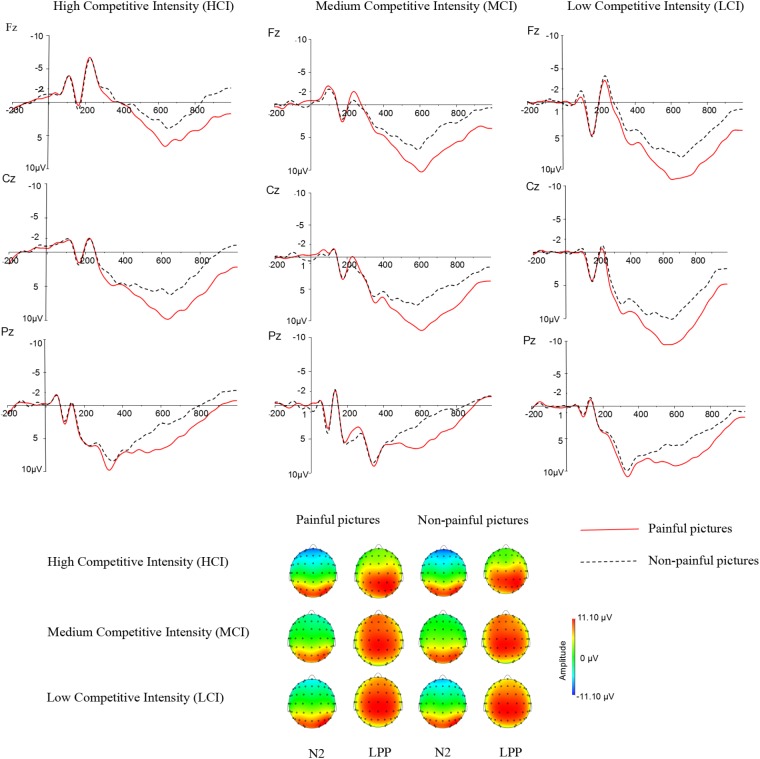
Average ERPs at Fz, Cz, and Pz for pain pictures and non-pain pictures under high competitive intensity (HCI), medium competitive intensity (MCI), and low competitive intensity (LCI) conditions, and the topographical map during 220–250 ms time windows (N2) and 350–600 ms time windows (LPP).

Previous studies have indicated that empathy for pain include an early emotional sharing component (N2) and a late cognitive evaluation component (LPP) (Luo et al., 2015; Cheadle, 2017). Moreover, grand averaged waveforms and topographical map of ERPs (see **Figure 2**) showed that those elicited by pain pictures and non-pain pictures in different competitive intensities were different and these differences were largest at frontal, central, and parietal sites. Thus, nine electrodes were selected for the following statistical analysis: F3, F4, Fz (frontal sites), C3, C4, Cz (central sites), P3, P4, Pz (parietal sites). Three-way ANOVA was conducted for N2 (220–250 ms) and LPP (350–600 ms) components. There was one between-group factor (competitive intensity: HCI, MCI, and LCI), and two within-group factors (stimulus: pain and non-pain pictures; electrode distribution: frontal, central, and parietal sites). The dependent variable was the mean amplitude for each component calculated from the frontal, central, parietal areas. Degrees of freedom for F-ratios were corrected according to the Greenhouse-Geisser method.

## Results

### Behavioral Performance

**Table 1** shows the mean scores and standard errors for each subscale of the IRI. One-way ANOVA showed that there was no difference between the three competitive intensity groups in PT, FS, EC, and PD subscales [PT: *F*(2,42) = 0.612, *p* > 0.05, η^2^ = 0.028; PD*: F*(2,42) = 1.085, *p* > 0.05, η^2^ = 0.049*;* EC: *F*(2,42) = 0.200, *p* > 0.05, η^2^ = 0.009; FS: *F*(2,42) = 0.767, *p* > 0.05, η^2^ = 0.035]. All participants correctly identified the pain cues on the recognition task when watching pain and non-pain stimuli during ERP recording sessions. All the participants’ answers to the questions “The hand in the picture belongs to whom?” and “What are your feelings when you see the opponent in pain?” were “the opponent” and “unpleasant”, respectively.

**Table 1:** Mean scores and standard error for the subscales of the IRI.

Scores	Interpersonal reactivity index (IRI)			
	PT	PD	EC	FS
HCI	11.20 (0.76)	7.27 (0.84)	16.73 (0.61)	9.47 (0.65)
MCI	10.73 (0.70)	9.53 (0.96)	16.53 (0.45)	11.13 (0.58)
LCI	12.13 (0.65)	7.97 (0.78)	17.23 (0.48)	10.29 (0.61)

*PT, perspective taking; EC, empathic concern; PD, personal distress; FS, fantasy.*

**FIGURE 3:**
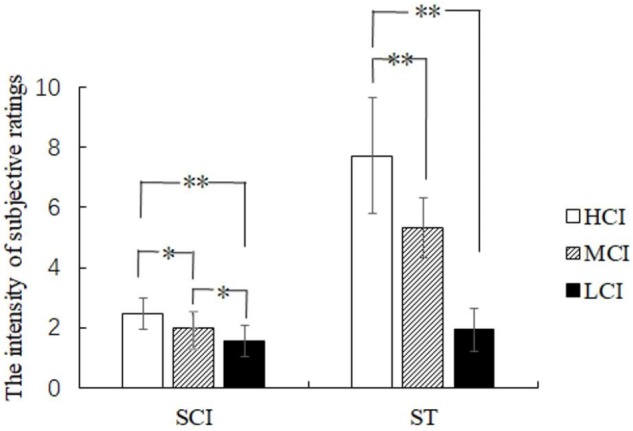
The rating scores of subjective competitive intensity (SCI) and subjective threat (ST). ^∗^*p* < 0.05 and ^∗∗^*p* < 0.01.

Subjective competitive intensity ratings were analyzed by one-way ANOVA and showed a significant difference in the three groups [*F*(2,41) = 16.646, *p* < 0.05, η^2^ = 0.448] (see **Figure 3**). Further analysis showed that the scores of the HCI group were significantly higher than that of the MCI group (*p* < 0.05) and the LCI group (*p* < 0.05), and the scores of the MCI group were significantly higher than that of the LCI group (*p* < 0.01). Moreover, one-way ANOVA revealed that the main effect of the threat level felt by the three groups was significant [*F*(2,41) = 13.127, *p* < 0.05, η^2^ = 0.390] (see **Figure 3**). The amount of threat experienced by the HCI group was significantly higher than that of both the MCI group (*p* < 0.01) and the LCI group (*p* < 0.01). However, there was no significant difference between the MCI and the LCI group (*p* > 0.05). Results thus indicate that the setting of the competitive intensity was effective. Moreover, the threat of HCI was found to be significantly stronger than that of MCI and LCI.

### ERP Results

For the N2 component (see **Tables 2**, **3**), ANOVA produced a significant main effect of electrode distribution [*F*(2,41) = 103.815, *p* < 0.05, η^2^ = 0.712]. The main effect of competitive intensity [*F*(2,42) = 2.805, *p* > 0.05, η^2^ = 0.118] and stimulus [*F*(1,42) = 1.196, *p* > 0.05, η^2^ = 0.028] were not significant. In addition, a two-way interaction between stimulus and competitive intensity was significant [*F*(2,42) = 5.536, *p* < 0.05, η^2^ = 0.209]. The simple effect analysis revealed that pain pictures elicited a more positive shift than non-pain pictures under the MCI (*t* = -5.118, *p* < 0.05) and LCI (*t* = 1.944, *p* < 0.05) conditions, but not under the HCI condition (*t* = 0.022, *p* > 0.05). A two-way interaction between competitive intensity context and electrode distribution was significant [*F*(4,84) = 4.110, *p* < 0.05, η^2^ = 0.164]. The simple effect of competitive intensity was significant at frontal site [*F*(2, 87) = 11.095, *p* < 0.05, η^2^ = 0.203] and central site [*F*(2,87) = 4.416, *p* < 0.05, η^2^ = 0.093], but not at parietal site [*F*(2,87) = 2.565, *p* > 0.05, η^2^ = 0.056]. In the frontal region and central region, a subsequent pairwise comparison showed that the LCI context elicited a smaller negative deflection than the MCI context *(p* < 0.01), and the MCI context elicited a smaller negative deflection than the HCI context (*p* < 0.05). No other interaction was found to be significant.

**Table 2:** Summary of ANOVA results of N2 (220–240 ms) and LPP (350–600 ms) with the electrode distribution (frontal, central, and parietal), stimulus (pain, non-pain) as the within-subject factors, and competitive intensity (HCI, MCI, and LCI) as the between-subject factor.

Effect	220–240 ms			350–600 ms		
	*F*	*P*	η_p_^2^	*F*	*P*	η*_p_*^2^
stimulus	1.196	0.280	0.028	89.104	0.000	0.680
electrode distribution	103.815	0.000	0.712	8.593	0.000	0.107
competitive intensity	2.805	0.072	0.118	3.155	0.053	0.131
electrode distribution × stimulus	0.721	0.489	0.017	0.044	0.957	0.001
stimulus × competitive intensity	5.536	0.007	0.209	1.223	0.305	0.055
electrode distribution × competitive intensity	4.110	0.004	0.164	4.075	0.005	0.163
electrode distribution × stimulus × competitive intensity	675.000	0.611	0.031	2.576	0.043	0.109

**Table 3:** Mean amplitudes (μV) and standard error at N2 (220–240 ms) and LPP (350–600 ms) shown by the three-way interaction of electrode distribution, stimulus, and competitive intensity.

Competitive intensity	220–240 ms		350–600 ms	
	Pain	Non-pain	Pain	Non-pain
High				
Frontal site	-5.07 ± 1.57	-4.91 ± 0.99	3.03 ± 0.91	2.23 ± 0.498
Central site	-0.24 ± 0.76	-0.19 ± 0.82	6.66 ± 0.46	5.61 ± 0.33
Parietal site	-0.25 ± 0.85	7.88 ± 0.61	6.94 ± 0.15	5.42 ± 0.26
Medium				
Frontal site	-0.35 ± 1.82	0.76 ± 1.23	8.47 ± 0.51	6.87 ± 0.41
Central site	1.46 ± 0.72	2.56 ± 1.02	8.95 ± 0.5	7.48 ± 0.26
Parietal site	6.10 ± 0.36	7.01 ± 0.75	6.91 ± 0.17	5.66 ± 0.13
Low				
Frontal site	-3.15 ± 1.32	-3.62 ± 1.45	7.53 ± 0.36	5.63 ± 0.59
Central site	0.20 ± 0.92	-0.27 ± 1.05	9.70 ± 0.27	7.91 ± 0.32
Parietal site	6.06 ± 0.34	5.70 ± 0.64	8.07 ± 0.32	6.66 ± 0.37

Repeated-measures ANOVA on the LPP component produced a significant main effect of electrode distribution [*F*(2,41) = 8.593, *p* < 0.05, η^2^ = 0.1701], stimulus [*F*(1,42) = 89.104, *p* < 0.05, η^2^ = 0.680] and a marginally significant main effect of competitive intensity [*F*(2,42) = 3.155, *p* = 0.053, η^2^ = 0.131] (see **Tables 2**, **3**). A two-way interaction between electrode distribution and competitive intensity [*F*(4,84) = 4.075, *p* < 0.05, η^2^ = 0.163] and a three-way interaction between electrode distribution, competitive intensity and stimulus [*F*(4, 84) = 2.576, *p* < 0.05, η^2^ = 0109] were found to be significant. Beyond that, no other significant interactions were observed. Focusing on the three-way interaction, significant two-way interaction was observed between competitive intensity and stimulus at frontal site [*F*(2,42) = 3.509, *p* < 0.05, η^2^ = 0.143], but not at central site [*F*(2,42) = 1.735, *p* > 0.05, η^2^ = 0.076] and parietal site [*F*(2,42) = 0.159, *p* > 0.05, η^2^ = 0.007]. In frontal region, simple effect of stimulus was significant under HCI (*t* = 2.790, *p* < 0.05), MCI (*t* = 4.339, *p* < 0.01), and LCI (*t* = 7.951, *p* < 0.01) conditions. Pairwise comparisons showed that pain pictures elicited larger LPP amplitudes than those elicited by non-pain pictures (*p* < 0.01) under HCI, MCI, and LCI condition, respectively. Moreover, simple effects of competitive intensity were found under both the pain condition [*F*(2, 42) = 6.885, *p* < 0.05, η^2^ = 0.247] and non-pain condition [*F*(2,42) = 5.330, *p* < 0.05, η^2^ = 0.202]. Subsequent multiple comparisons showed that the amplitudes of LPP elicited under HCI condition were significantly smaller than those elicited under MCI (*p* < 0.01) and LCI (*p* < 0.01) conditions. However, no significant differences between the LPP amplitudes elicited in MCI and LCI condition were found.

## Discussion

Previous studies have investigated empathic response to other’s pain within a competitive context (Koban et al., 2012; Cui et al., 2016). The present work extends previous research by examining the neural processes underlying responses to viewing others in painful and non-painful situations within different kinds of competitive (HCI, MCI, and LCI) context. We found that empathic responses were modulated by competitive intensity. Specifically, we observed early N2 differentiation between painful and no-painful situations over the frontal area under MCI and LCI conditions. In contrast, no such early ERP response was detected under HCI condition. Moreover, we observed a pattern of pain and no-pain differentiation for the late LPP over the frontal and centro-parietal regions under HCI, MCI, and LCI condition. These results indicated that pain empathic response is down-regulated under HCI condition at early N2 stage.

Our behavioral results showed that participants identified the painful situation of the opponents during ERP recording sessions and felt unpleasant when watching the opponents in pain. This suggests that empathic response but not schadenfreude was induced by the opponents’ pain in our study. Societal expectation and the amount of reward may contribute to the affective response. First, participants try to fit a role based on society expectation because kindheartedness is advocated by intellectuals in Chinese culture. Second, the amount of reward is too little to induce schadenfreude. Although subjective competitive intensity ratings showed a significant difference in the three groups (HCI group > MCI group > LCI group), there was no significant difference between the threat level felt by the MCI and the LCI group. However, the threat of HCI was significantly stronger than that of MCI and LCI. The results indicate that high competitive intensity context is more likely to modulate the neural underpinnings of pain empathy response due to its bigger threat.

According to previous research results, the frontocentral N2 component is thought to reflect aspects of response conflict and response inhibition (Botvinick et al., 2004; Kerns et al., 2004; Luo et al., 2013). The larger N2 amplitudes, the higher level of conflict and inhibition will be. People tend to vicariously resonate with the pain of others (Lamm et al., 2011). However, pain empathy is not obligatory (Cameron et al., 2017). The “threat value of pain” hypothesis demonstrates that processing of another’s pain also may be associated with a threat, which informs us of potential harm and promotes self-protective response (Williams, 2002; Yamada and Decety, 2009; Ibáñez et al., 2011). Empathic response is other-oriented while protective response is self-oriented. Thus, these two responses are conflicting and the inhibition of self-protective response is important during the empathic task. In our study, N2 amplitudes in HCI condition were significantly larger than that of in MCI condition, and N2 amplitudes in MCI condition were significantly larger than that of in LCI context. The results show that as competitive intensity increase, the conflict and inhibition is getting stronger and stronger. More importantly, we found neutral pictures elicited more negative deflections than painful pictures only in MCI and LCI condition, but not in HCI condition. A highly competitive intensity context creates a negative and threatening atmosphere, which in turn triggers a system of threat-detection and induces negative emotion. Individuals who are in bad moods have difficulty in focusing on others’ painful situations (Baron-Cohen et al., 2004). One possible explanation is that the HCI context may induce a greater sense of threat and lead to a negative emotion, which forces people to ignore other’s pain. Therefore, the bottom-up processing of participants’ perception of pain experienced by their opponent is absent. Our results are consistent with previous studies which have demonstrated that the automatic process of empathy (N2) can be affected by competitive context (Cikara et al., 2014; Cui et al., 2016).

The LPP component is considered to reflect a facilitated process attention to emotionally relevant or motivationally salient stimuli (Schupp et al., 2000, 2003; Hajcak et al., 2010; Kiat and Cheadle, 2017). We found that LPP amplitudes elicited in HCI condition were significantly smaller than in MCI and LCI conditions. Our behavioral data showed that the subjective threat level felt by the HCI group was significantly stronger than by the MCI group and the LCI group. One possible explanation is that people feel more threats in HCI condition, which in turn leads to less attention paid to painful stimuli. In this case, painful stimuli elicited the smallest LPP amplitudes in HCI context. In addition, our LPP results showed that pain stimuli elicited larger amplitudes than non-pain stimuli under all three competitive contexts. Previous studies suggest that, compared with neutral stimuli, negative stimuli would recruit more physiological and psychological resources due to the evaluation of evolutionary importance (Yuan et al., 2007). Thus, negative stimuli elicit larger LPP amplitudes than neutral stimuli (Luo et al., 2013, 2015). Our results are consistent with these previous studies and indicate that empathic response could be found within three competitive intensity contexts at late cognitive controlled stage.

In conclusion, the current ERP study provides new neuroscientific insights into how differing levels of competitive intensity affect the ability to experience empathy for pain. Previous studies have suggested that empathy involves both bottom-up and top-down information processing (Decety and Lamm, 2006). The former is automatic and allows individuals to experience similar emotional states to others, whereas the latter is equivalent to an elaborative process that reflects the integration of cognitive control and reappraisal (Dennis and Hajcak, 2009; DeCicco et al., 2012). Our results suggest that, under condition of high intensity competition, sensory processing elicited by the perception of pain during the automatic emotional sharing stage (N2) is absent and pain empathy response at the late cognitive evaluation stage (LPP) is less obvious. Our results allow a better understanding of the mechanism underlying the effect of competition on pain empathy. One limitation is that we have no control group, which can be compared with three groups to observe the empathy for others’ pain between different competitive intensity contexts and control context, and then to control the group as a reference to understand the empathy of pain in different competitive intensity contexts separately. Another limitation is that the present research only focuses on empathy in relation to physical pain, it overlooks other ways in which people readily empathize with others in everyday life such as social pain and emotional suffering (Zaki et al., 2009; Masten et al., 2011; Rameson et al., 2012). Thus, for the purposes of ecological validity, future studies would benefit from examining how these other forms of empathy might be influenced by modulating levels of competitive context.

## Ethics Statement

This study was carried out in accordance with the recommendations of the Academic Committee of South China Normal University with written informed consent from all subjects. All subjects gave written informed consent in accordance with the Declaration of Helsinki. The protocol was approved by the Academic Committee of South China Normal University.

## Author Contributions

PL designed the experiments, analyzed the data, and wrote the paper. YP analyzed the data and wrote the paper. BL, JJ, MZ, and SY collected the data. XZ designed the experiments.

## Conflict of Interest Statement

The authors declare that the research was conducted in the absence of any commercial or financial relationships that could be construed as a potential conflict of interest.
